# The Combined Effects of Urine Zinc, Cadmium, Mercury, Lead, and Copper on Endometrial Cancer Staging

**DOI:** 10.3390/ijerph22020245

**Published:** 2025-02-10

**Authors:** Issah Haruna, Russell R. Broaddus, Andrew B. Gladden, Kiran Subedi, Emmanuel Obeng-Gyasi

**Affiliations:** 1 Department of Built Environment, North Carolina A&T State University, Greensboro, NC 27411, USA; 2 Environmental Health and Disease Laboratory, North Carolina A&T State University, Greensboro, NC 27411, USA; 3 Department of Pathology and Laboratory Medicine, The University of North Carolina at Chapel Hill, Chapel Hill, NC 27599, USA; 4 Pathobiology and Translational Science Graduate Program, The University of North Carolina at Chapel Hill, Chapel Hill, NC 27599, USA; 5 Analytical Services Laboratory, College of Agriculture and Environmental Sciences, North Carolina Agricultural and Technical State University, Greensboro, NC 27411, USA

**Keywords:** environmental pollutants, EC, essential element, heavy metals, Bayesian kernel machine regression, linear regression univariate and bivariate exposure-response

## Abstract

Endometrial cancer (EC) is a growing public health concern. This secondary data study of a case series leveraged existing samples and data to explore the potential link between exposure to heavy metals/essential elements and stage of EC. We analyzed urine samples from women with EC, measuring levels of toxic metals (cadmium, mercury, and lead) and essential elements (zinc and copper). Our findings revealed that higher levels of mercury, cadmium, and lead are associated with more advanced EC stages. Conversely, zinc showed a protective effect, potentially mitigating EC stage progression. Copper levels did not show a clear association with EC stage. These results highlight the potential impact of environmental exposures on EC stage and the crucial need for advanced statistical methods to understand the combined effects of these pollutants on health and the need for public health interventions. Further research is needed to explore the mechanisms by which these metals influence EC stage and long-term outcomes.

## 1. Introduction

Endometrial cancer (EC) is a malignancy of the inner epithelial lining of the uterus with an increasing stage and disease-associated mortality worldwide and comprises distinct histological subtypes and molecular phenotypes [1]. According to the American Cancer Society, EC is the most common gynecologic cancer and the fifth most common cancer among women in the United States. They estimated that approximately 67,880 new cases of uterine cancer would be diagnosed, and 13,250 women would die from endometrial cancer (EC) in 2024 [2,3]. More recent data from the American Cancer Society estimate 69,120 new cases and 13,860 deaths from EC in the United States in 2025 [4].

Annual mortality has been especially increasing in Black patients [5]. Broadly, EC can be divided into endometrioid or non-endometrioid cancer, identified using microscopic examination by a pathologist. Molecular subtypes include copy number high, copy number low, *POLE*-mutated, and microsatellite-instability (MSI) high, which represent the most comprehensive genomic analysis of endometrial carcinoma (EC) by the Cancer Genome Atlas (TCGA) [6]. Many of the non-endometrioid ECs, such as serous carcinoma, are copy number high with *TP53* mutations and are typically associated with worse patient survival [7].

The International Federation of Gynecology and Obstetrics (FIGO) classifies uterine cancer into four stages based on the extent of cancer progression. Stage I is confined to the uterus, whereas Stage II indicates cancer has spread to the cervix. Stage III involves further spread to the vagina, ovaries, and/or lymph nodes. Stage IV represents the most advanced stage, with cancer metastasizing to the urinary bladder, rectum, or distant organs such as the lungs or bones. This staging system provides a framework for understanding disease progression and guiding treatment decisions [8].

The concept of the exposome encompasses the totality of environmental exposures an individual encounters throughout their lifetime, from conception to death. These exposures include a wide range of chemical, biological, and physical agents, as well as lifestyle factors [9,10]. In environmental health research, exposure to individual metals and elements such as lead (Pb), cadmium (Cd), mercury (Hg), zinc (Zn), and copper (Cu) are often studied in isolation to assess their specific impacts on health. Studies typically do not account for the combined effects of multiple exposures in human subjects. However, in real-world scenarios, individuals are exposed to complex mixtures of these and other environmental agents simultaneously. The combined effects of Pb, Cd, Hg, Zn, and Cu, for example, can lead to interactions that may amplify or mitigate the individual effects observed when these metals are studied in isolation [11].

Research on the combined effects of multiple exposures is challenging due to the complexity of interactions and the need for sophisticated analytical methods. However, it is crucial for a comprehensive understanding of the exposome and its impact on human health. Essential metals, such as zinc, selenium, and copper, play vital roles in numerous physiological processes, including enzymatic activity, DNA repair, and antioxidant defense mechanisms. These elements are required in trace amounts for maintaining cellular function and overall health. However, when present in excessive concentrations, essential metals can cause toxicity by disrupting cellular homeostasis and promoting oxidative stress, inflammation, and DNA damage [12,13,14]. Such toxic effects can contribute to the development and progression of diseases, including cancer [14].

Conversely, toxic metals like cadmium, arsenic, lead, and mercury are not required for physiological processes and are harmful even at low levels of exposure. Investigating the interplay between essential and toxic metals is critical, as imbalances may lead to complex synergistic, antagonistic, or additive effects. Understanding these interactions is key to better assessing risks and developing targeted public health interventions to mitigate exposure-related health consequences, such as those associated with EC. Heavy metals are one of the most studied groups of environmental contaminants with several elements evidenced to display toxic effects on reproductive tissues, organs, and systems [15]. Metals are suspected to induce genotoxicity through multiple mechanisms. However, previous authors proposed that the initiation and progression of cancer occurs through the interference with cellular redox regulation, which causes oxidative stress, a state in which the increased production of reactive oxygen species (ROS) is not sufficiently compensated by the antioxidant protection of the body, which is followed by DNA damage [16].

The evaluation of metal status in female reproductive tissues and determination of factors potentially influencing this status is of particular interest given that some elements support normal reproductive function when in their right proportion, whereas others are recognized to induce serious impairment [17].

Particular attention is drawn to an emerging class of so-called metalloestrogens with growing evidence suggesting that they possess the ability to bind estrogen receptors and subsequently give rise to estrogen agonist responses [18]. Metalloestrogens are a class of inorganic metals or metalloids that can affect the gene expression of human cells that respond to estrogen. Their effects are related to the physiological function of estrogen because they have shown affinity for estrogen receptors and can mimic estrogen, leading to the activation the receptors. Hence, they are considered very harmful and potentially linked to cancer [19]. Epidemiologically, they have been potentially implicated in estrogen-dependent diseases such as breast cancer and EC as well as endometriosis [18,20]. However, the literature on the cumulative effect of essential elements and toxic metals is limited [17]. Hence, an assessment of the combined effect of essential elements and toxic metals is, therefore, crucial. Consequently, the purpose of this study was to determine the association between essential elements (Zn and Cu) and heavy metals (Pb, Cd, and Hg) with EC stage. The potential antagonistic or synergistic effects of combined trace and heavy metal exposure on EC stage were also examined.

## 2. Materials and Methods

### 2.1. Patient Population

This secondary data case series study leveraged data and samples from an already approved study that underwent institutional review boards at both the University of Texas MD Anderson Cancer Center (LAB 02-188) and the University of North Carolina School of Medicine (IRB 22-1802). Our study examined a retrospective cohort of 266 patients undergoing hysterectomy surgery for EC at the University of Texas MD Anderson Cancer Center between 2000 and 2020. All 266 patients were included in the data analysis.

Patients were eligible for inclusion if both urine samples and formalin-fixed, paraffin-embedded EC tumor tissue were available. The study cohort included cases with both endometrioid and non-endometrioid histology. EC histology and stage were determined with microscopic analysis conducted by gynecological pathologists. Clinical and pathological variables were obtained from pathology reports and electronic medical records. Urine samples (5 mL) were collected at the time of surgery via sterile catheterization of the urinary bladder, divided into 1 mL aliquots, and stored at −80 °C until the analysis of heavy and essential metals.

The EC cohort from this study is derived from a large NCI-designated cancer center. A comparison with published data from a national epidemiological analysis of 161,513 EC patients indicates that the MD Anderson cohort is comparable in terms of age at diagnosis, the proportion of endometrioid versus non-endometrioid histology, and stage distribution. The only notable difference is a higher proportion of women with grade 2 and 3 endometrioid carcinomas in the MD Anderson cohort.

In this study, the term “early” refers to uterine (endometrial) cancer stages I and II, while “late” encompasses stages III and IV. Although stage II is sometimes categorized as intermediate, they are collectively referred to as “early” for the purpose of analysis. In summary, the final dataset for this study included a total of 266 patients who met the inclusion criteria and were used for data analysis.

### 2.2. Urine Metal/Essential Element (Zn, Cu, Pb, Cd, and Hg) Measurements

Inductively coupled plasma optical emission spectroscopy (ICP-OES) analysis of patient urine was performed using an Optima 8300 ICP-OES (Perkin Elmer, Inc., Shelton, CT, USA). Prior to analysis, aliquots of urine samples were filtered through 25 mm cellulose acetate syringe membrane filters (45 μm pore size) into clean 15 mL conical plastic tubes to reduce interferences from particles. One milliliter of the filtered urine sample was placed in a polypropylene tube with an internal standard solution containing 10 ng of rhodium (Rh) and thallium (Tl). To this mixture, 0.3 mL of concentrated nitric acid was added. The solution was then diluted using ultrapure water.

The concentrations of heavy metals and essential elements (Hg, Cu, Zn, Cd, and Pb) were measured using ICP-OES. A commercial mixed standard solution (High Purity Standards, Charleston, SC, USA) was used, with appropriate dilution for calibration, and internal standardization was applied. Tl was used as the internal standard for Pb determination, and Rh was used for the other metals. Spectral interference was corrected based on the oxide formation rate experimentally obtained.

The limits of detection (LOD) for metals in urine samples were determined using inductively coupled plasma optical emission spectroscopy (ICP-OES). The LODs were calculated using the three-sigma (3σ) method, in which the standard deviation (σ) of blank sample intensities was multiplied by a factor of three to establish the lowest reliably detectable concentration. Based on this approach, the LOD values for each metal were as follows: zinc (Zn)—76.49 µg/L, cadmium (Cd)—3.42 µg/L, lead (Pb)—3.74 µg/L, copper (Cu)—3.42 µg/L, and mercury (Hg)—5.00 µg/L. These values were derived by analyzing the variability in blank emission intensities and converting the results using calibration curves.

To ensure accuracy and reproducibility, quality control measures such as matrix-matched standards, internal controls, and calibration verification were implemented throughout the analytical process.

### 2.3. Statistical Analysis

#### 2.3.1. Descriptive Statistics

We began our study by analyzing descriptive statistics to characterize the clinical and pathological features of EC patients and to examine the relationships between heavy metal exposures and stage of EC. This study is not a case–control study but instead includes a cohort of patients all diagnosed with EC. The clinical classification of patients into endometrioid and non-endometrioid subtypes was based on pathological analysis of tumor histology, as determined by gynecological pathologists. Continuous variables, such as age and BMI, were summarized as means with standard errors (SE) and compared across EC subtypes (endometrioid vs. non-endometrioid) using *t*-tests. Categorical variables, including race, stage category, and myometrial invasion, were presented as frequencies and percentages and compared using chi-square tests. This approach allowed us to explore differences in clinical and pathological features across subtypes rather than designating one as a control group.

Although we used means for preliminary descriptive statistics to provide a straightforward measure of central tendency and facilitate comparisons between groups, due to their limitations, including sensitivity to outliers, we employed robust statistical methods, such as Spearman correlation. Spearman correlation ranks data rather than relying on raw values, making it resistant to the influence of outliers and suitable for non-normal data. Additionally, biserial Pearson correlation was used for binary variables to ensure appropriate handling of the data’s structure. These approaches provided a comprehensive and reliable assessment of the relationships between pollutants and EC characteristics while mitigating potential biases from outliers or skewed distributions. This combination of methods ensured that our analyses were both robust and meaningful, addressing concerns about the limitations of mean-based descriptive measures.

#### 2.3.2. Bayesian Kernel Machine Regression (BKMR)

In this study, we used Bayesian kernel machine regression (BKMR) utilizing the Markov chain Monte-Carlo (MCMC) sampling approach, as described by Bobb et al. [11]. To gain a better understanding of the interplay between these essential elements and toxic metals and the outcome of interest (EC stage), we calculated high-dimensional exposure-response functions, denoted as h(z), at various intervals. This was accomplished while holding the other impacting variables at their median [21].

The BKMR model stood out for its graphical interpretation function in our analysis. This capability enables a comparative investigation of the impacts of toxic metals and essential elements, both collectively and individually, and compares the outcome observed at certain exposure quantiles to those at median exposure levels. It also emphasizes the distinct association between each metal and element with EC stage, considering the constant median values of other exposures. This analytical technique enabled a more comprehensive understanding of the nuanced individual and cumulative effects of Zn, Cu, and toxic metals such as Pb, Cd, and Hg on EC stage.

Analysis was conducted with R (version 4.2.3; R Foundation for Statistical Computing, Vienna, Austria) [22]. The level of significance was set at 0.05 and adjusted for the covariates, age, BMI, and ethnicity.

## 3. Results

### 3.1. Patient Demographics and Clinical Characteristics

This study examined a retrospective cohort of 266 patients undergoing hysterectomy surgery. Out of the total patients, 86.1% were diagnosed with endometrioid EC, while 13.9% were with non-endometrioid EC (Table 1). Consistent with usual EC patient populations, most of the patients had early-stage endometrioid cancers, and patients with endometrioid cancers had significantly higher BMI compared to patients with non-endometrioid cancers. The ethnic distribution showed that the majority were white (69.0%), followed by Hispanic (21.4%) and Black (9.6%). This race distribution is consistent with the findings by [23] and characteristic of most tertiary care cancer centers in the United States.

The stage of diagnosis is one of the most important determinants of EC patient survival. Overall, for each metal, there was no significant difference in mean metal levels and EC stage (Table 2).

### 3.2. Linear Regression Analysis of Toxic Metals and Essential Elements in Relation to EC Stage

We performed linear regression analysis to assess the association between metals exposure and EC stage, after controlling for race, BMI, and age at diagnosis. The coefficient from Table 3 indicates that exposure to the essential elements (Zn and Cu) was negatively associated with EC stage, whereas exposures to the toxic heavy metals (Hg, Cd, and Pb) were positively associated with EC stage. However, the associations did not achieve statistical significance, as evidenced by *p*-values exceeding the 0.05 threshold.

### 3.3. Correlation Analysis of Toxic Metals and Essential Elements with EC Stage

**Figure 1:**
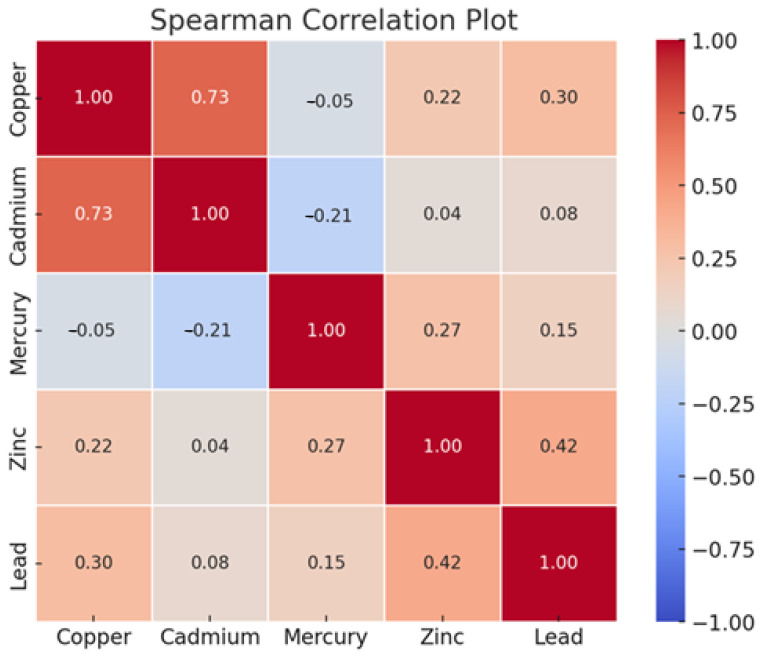
Spearman correlation of toxic metal exposure and essential elements.

Figure 1 displays the Spearman correlation analysis of the study’s exposure variables. The results show a strong positive relationship among all the metals, except Hg and Cd, which had a weak negative correlation.

**Figure 2:**
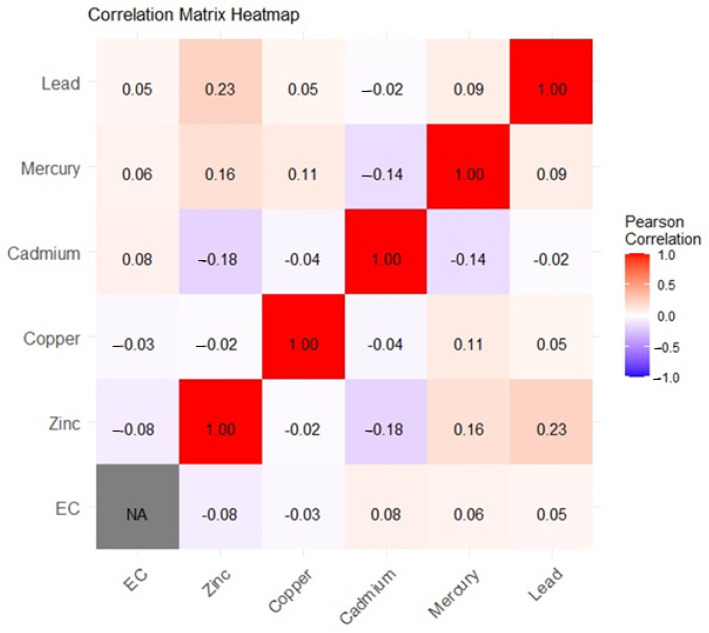
Pearson correlation matrix heatmap among exposure variables and EC stage. Red represents a positive correlation, grey indicates no correlation, and blue represents a negative correlation.

Figure 2 presents a biserial Pearson correlation matrix heatmap of metal exposure and EC stage. The results reveal a positive correlation among most the heavy metal mixtures and a negative correlation between some the essential elements and heavy metals and EC stage. The results also depict a negative correlation between the essential metals (Cu and Zn) and EC stage and a weak positive correlation between the heavy metals Pb, Hg, and Cd and EC Stage.

### 3.4. Bayesian Kernel Machine Regression (BKMR) Analysis of Toxic Metals and Essential Elements

BKMR is a flexible statistical method used to assess the joint effects of multiple environmental exposures on health outcomes. It allows for the examination of complex, non-linear relationships and interactions among multiple pollutants. By modeling the joint impact of these exposures, BKMR provides a more comprehensive understanding of how various environmental factors collectively influence health.

#### 3.4.1. Posterior Inclusion Probabilities of Metals and Elements in EC Stage

The posterior inclusion probability (PIP) table highlights the relative importance of five variables—zinc, copper, cadmium, mercury, and lead—in their association with EC stage. Zinc (PIP = 0.326) and copper (PIP = 0.272) exhibit the highest probabilities, indicating their stronger potential roles in influencing the disease. In contrast, cadmium (PIP = 0.152), lead (PIP = 0.122), and mercury (PIP = 0.084) demonstrate weaker evidence for inclusion in the model, suggesting a lower likelihood of significant impact. These findings underscore the varying degrees of influence these elements may have on EC stage. The results are presented in Table 4.

The hierarchical analysis examines the relationship between metals/elements and the EC stage using a group-wise Bayesian framework. Variables are categorized into two groups based on shared characteristics or exposures. Group 1, consisting of zinc and copper, shows a higher group-level posterior inclusion probability (groupPIP = 0.655), indicating these elements collectively have stronger relevance to EC stage. Within this group, zinc exhibits the highest conditional posterior inclusion probability (condPIP = 0.517), suggesting it is the most influential individual variable, closely followed by copper (condPIP = 0.483).

In Group 2, which includes cadmium, mercury, and lead, the overall groupPIP is lower (0.457), reflecting reduced collective relevance. However, individual condPIP values reveal notable variation. Lead (condPIP = 0.682) emerges as a significant contributor, suggesting its potential importance despite the group’s lower overall relevance. In contrast, cadmium (condPIP = 0.278) shows moderate importance, and mercury (condPIP = 0.039) exhibits minimal evidence for inclusion in the model.

This hierarchical approach (Table 5) highlights distinct group-level and individual contributions, with zinc and lead identified as key metals requiring further investigation for their roles in EC risk.

#### 3.4.2. Univariate Exposure–Response Analysis of Metals and Elements in EC Stage

**Figure 3:**
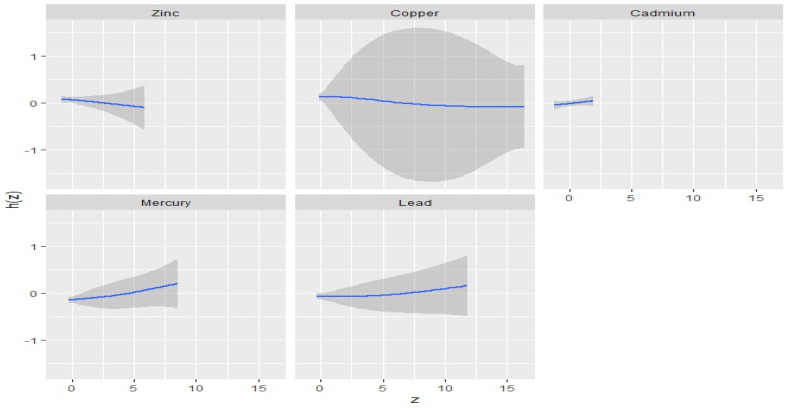
Univariate exposure–response functions and 95% credible intervals for the association between single pollutant exposure when other pollutant exposures are fixed at the median. Results adjusted for BMI, age, and ethnicity.

The univariate approach within BKMR visually examines the individual effects of Zn, Cu, Hg, Cd, and Pb on the outcome of interest, in this case, EC. Figure 3 illustrates the impact of each metal on EC stage when the other metals are held at their median levels and covariates are kept constant. The plot suggests a linear increasing effect of Hg, Pb, and Cd on EC stage, indicating that higher levels of these metals are associated with higher stages of EC.

In contrast, the Zn panel in Figure 3 shows a slight decrease in its effect on EC stage, while Cu’s plot remains relatively flat. This suggests that, within the range of exposures examined, variations in Cu exposure do not substantially impact EC stage. Overall, these findings highlight the differential impact of each metal on EC stage, emphasizing the importance of considering individual and combined effects.

#### 3.4.3. Bivariate Exposure–Response Analysis of Pollutant Pairs and EC Stage

**Figure 4:**
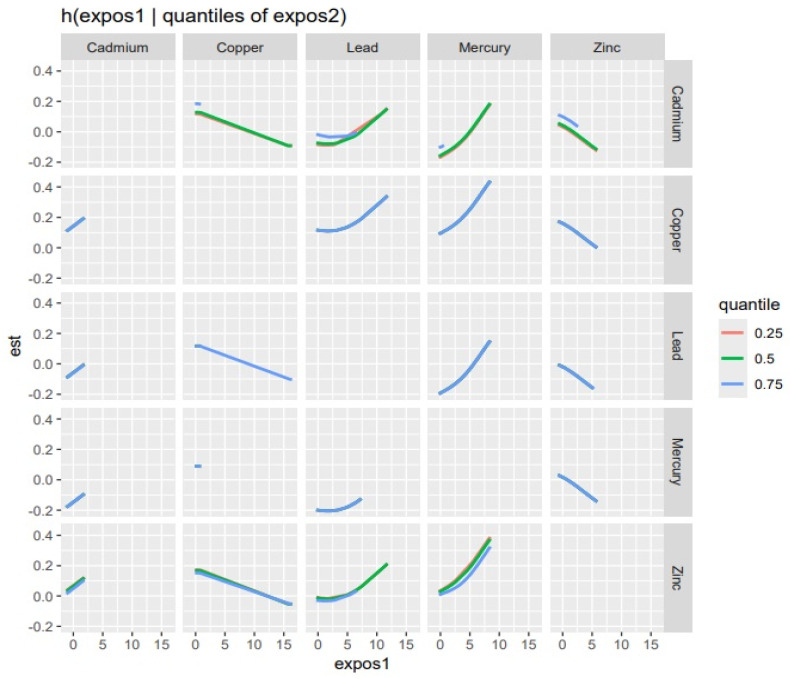
Bivariate exposure–response function of every two exposures with EC stage. The predictor–response function is investigated with varying quantiles of the second predictor, while other predictors are fixed at the median. Results adjusted for age, ethnicity, and BMI.

The bivariate association was subsequently investigated by looking at the effects of pollutant pairs on EC stage (Figure 4). The analysis investigated the relationship between specific metal and EC stage by fixing the second pollutant at different quantiles of 0.25 (red line), 0.5 (green line), and 0.75 (blue line) while keeping the other pollutant at the median. These models were adjusted for the covariates of interest (age, BMI, and ethnicity). The *x*-axis, designated “expos1”, depicts the levels of one exposure, whereas the *y*-axis, labeled “est”, displays the estimated effect on EC stage. Each column of the graph represents a different exposure, denoted as “expos1”, with each row being “expos2”.

Interaction Effect: Each plot depicts how the relationship between an exposure “expos1” varies with the quantiles of a second exposure, “expos2” on EC stage. The three lines within each plot represent the 0.25, 0.50, and 0.75 quantiles of “expos2”, as shown by the color legend.

The interpretation of the graph as depicted by each pollutant is as follows:

Cadmium (as expos1): The plot for Cd reveals slightly increased relationships with EC stage at different quantiles for Cu, Pb, Hg, and Zn (at the 0.5 and 0.75 quantile), which shows that higher levels of Cd could have an increased effect on EC stage.

Copper (as expos1): Cu’s interaction plot reveals a decreased association with EC stage at the 0.5 and 0.75 quantile for Cd and Zn. Additionally, the 0.75 quantile Pb is related to a decreased effect of the EC stage.

Zinc (as expos1): Zn’s interaction plot also reveals a negative slope (decreased association) with EC stage, indicating that an average or adequate level even at the 0.5 and 0.75 quantiles for Cd, Zn, and Cu, Pb have a decreased effect on EC stage.

Pb and Hg (as expos1): The bivariate interaction plot of Pb and Hg depicts a positive slope at all quantiles of Cd, the 0.5 quantile of Cu, and the 0.5 and 0.75 of Zn. This reveals that increasing levels of Pb and Hg can increase the effect on EC stage.

#### 3.4.4. Single-Variable Effects of Metals and Elements on EC Stage

The single-variable effect aids in understanding the impact of a single predictor at various quantiles, allowing us to analyze its contribution to the total risk of increased EC stage.

**Figure 5:**
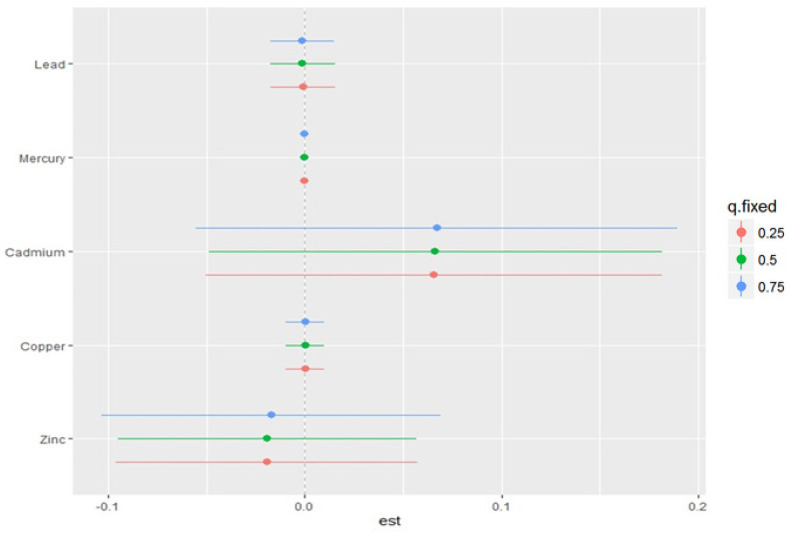
Single-variable effect of essential elements and toxic metals exploring changes of each exposure from the 25th to 75th percentile, while fixing all the other exposures at specific quantiles—0.25 (red), 0.50 (green), and 0.75 (blue). Results adjusted for age, ethnicity, and BMI.

Figure 5 depicts the single-variable effects of pollutants on EC stage at the 25th (red), 50th (green), and 75th (blue) quantiles, which suggest that Cd is associated with higher values of the h function, a flexible function that takes multiple pollutants and combines them in a way that captures the complex and potential relationship between the various metal and EC stage. Overall, the plot and quantiles demonstrate how the link between each metal and EC stage varies depending on the exposure distribution of the pollutants. It also gives insight into the interaction of each pollutant.

#### 3.4.5. Overall Effects of Toxic Metals and Essential Elements on EC Stage

**Figure 6:**
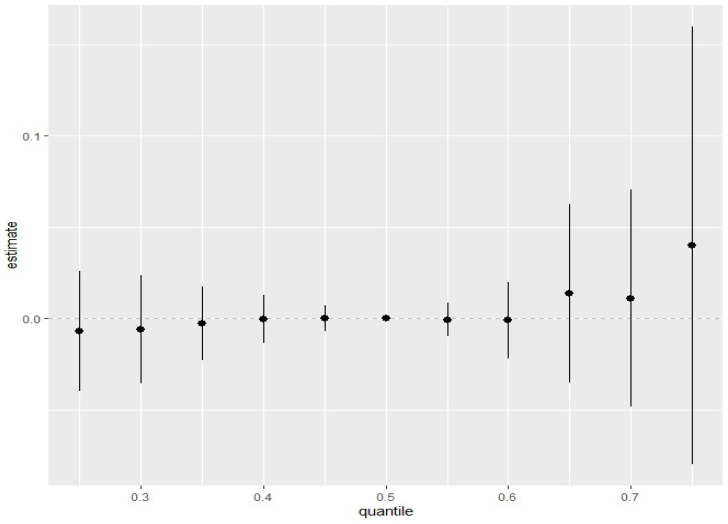
The summary of the overall risk (dots) with 95% credible intervals of the exposures (essential elements and toxic metals) on EC stage explored at various quantiles (from 0.25 through 0.75) compared to the median quantile (0.5). Results adjusted for BMI, age, and ethnicity.

Figure 6 depicts the overall effect of all multipollutant mixtures on EC stage. The exposures are fixed at different quantiles ranging from the 25th quantile to the 75th quantile in five-point increments, with the 50th quantile (median value) serving as the point of comparison for the exposures. As shown in Figure 6, we found that the estimated risk of higher EC stage increased with a simultaneous increase in all five pollutants, from the 25th quantile to the 75th quantile, as compared to when all pollutants are at their 50th quantile (median values). This indicates a positive joint effect of the pollutant mixtures, particularly when all five pollutants are within their 60th and 75th quantile, although the credible intervals were larger at these values.

## 4. Discussion

EC is the second most predominant gynecological malignancy. Previous authors have posited a wide range of overarching factors known to increase the rate of ECs, including obesity, aging, late menopause onset, chronic anovulation, and polycystic ovarian syndrome [24].

One of the unique aspects of our study is the analysis of patient urine to characterize the levels of toxic metals (Pb, Cd, and Hg) and essential elements (Cu and Zn). This approach sets our study apart from others that have primarily focused on analyzing heavy metal levels in serum or tumor tissue. Urine analysis provides a different perspective on metal exposure and excretion, potentially offering insights into the body’s handling and detoxification processes of these metals.

Comparing our data with studies that analyzed heavy metals in serum or tumor tissue reveals interesting differences and similarities. Serum analysis typically reflects recent exposure and circulating levels of metals, whereas tumor tissue analysis can indicate long-term accumulation and potential local effects. Urine analysis, on the other hand, gives us a snapshot of the metals being excreted, which may correlate with both acute and chronic exposure levels. The ability to capture these different aspects of metal exposure and metabolism highlights the robustness and comprehensive nature of our study.

Endometrial diseases such as adenomyosis, endometrial polyps, and uterine fibroids are the major causes of infertility among women that could chiefly be attributed to hormonal and metals (essential elements and toxic metals) imbalance. However, studies on the combined effects of essential elements and toxic metals on EC and the risk of developing the disease are limited. The current study investigated the combined effects of essential elements and toxic metals on EC stage.

PIP results demonstrated that essential elements like zinc and copper were more important predictors of EC stage compared to toxic metals. Among the toxic metals, lead and cadmium showed the highest impact, while mercury had a minimal influence. Zinc emerged as the most significant within the essential elements group.

Broadly, these findings highlight the dual role of essential elements. While critical for physiological functions at normal levels, they can become harmful at excessive concentrations. Toxic metals, on the other hand, are consistently harmful, contributing to oxidative stress, cellular dysfunction, and carcinogenesis. Understanding the nuanced interplay between essential and toxic metals is crucial for developing targeted interventions to mitigate environmental risks for EC.

Our BKMR analysis of the bivariate exposure–response revealed that Pb and Hg have a significant impact on EC stage. The single-variable effect of Cd likewise exhibited an increased association with EC stage, which is consistent with the findings of Zhang et al. [25] demonstrating that Cd contributed significantly to cancer. Cd has been classified as a class I carcinogen according to IARC [26] and can contribute to the development of cancer by inducing oxidative stress [27]. Bioaccumulation of different toxic metals affect the functionality of cells, such as proliferation and differentiation, and induce oxidative stress through the generation of reactive oxygen species (ROS). The resulting oxidative stress will induce cellular damage [28]. Humans are protected from chronic exposure to low Cd concentrations by the presence of metallothioneins (MTs), a family of ubiquitous small cysteine-rich proteins, whose specific function is to regulate the metabolism of Zn. MTs play an important role in protection against ion toxicity from several heavy metals (such as Cd, Hg, and Pb), DNA damage, and oxidative stress [27]. Regarding the impact of heavy metals on EC, there is evidence in the literature suggesting that MTs may be altered in EC [29,30,31]. MTs can bind to heavy metals, reducing their toxicity and mitigating oxidative stress, which is a known factor in cancer progression [32].

Essential trace metals such as Cu, Zn, Fe, and Se are important cofactors of a variety of enzymes in the human body and play important roles in the inhibition of the production of ROS and free radicals, immunological functions, cell growth, and differentiation to maintain normal body physiology and homeostasis [13]. Again, these essential elements have the potential to enhance overall immunity, promote DNA damage repair, exhibit an anti-tumor role, and play a role in cancer suppression, which can contribute to cancer treatment and prevention when in their right proportions [33,34]. These findings are consistent with our present study, which revealed that Cu and Zn are both significantly negatively correlated or associated with EC stage as depicted in our Pearson correlation matrix heatmap (Figure 2), univariate exposure–response functions (Figure 3), bivariate exposure–response function plot (Figure 4), and the single-variable effect function plot (Figure 5). This notwithstanding, excess levels of essential metals and imbalances in their ratios (for instance, Cu/Zn ratio) may contribute to the development of cancer by inducing peroxidation stress, abnormal cell proliferation, and cell injury [12,35,36].

The combined effect of mixed exposure to all five metals (essential and toxic metals) (Figure 6) showed that the risk of developing EC can be significantly increased when the concentration of all the metals is higher than the 60th quantile. The presence of essential elements likely offered some protective effect as metals alone in combination may have resulted in a much steeper positive curve.

### Limitations

This study explored the use of urine samples for detecting and measuring various essential elements and toxic heavy metals. While urinalysis provides a convenient and non-invasive approach for assessing metal levels, it may not fully capture the long-term body stores or concentrations in specific tissues, such as the endometrium. Furthermore, the validity of urinary metal levels as an indicator of body burden varies by metal. For example, while urinary levels are reliable for cadmium (Cd), blood levels are considered more accurate markers for metals such as lead (Pb) and mercury (Hg), particularly organic forms like methylmercury. The absence of blood metal analysis in our study is a limitation, as blood samples could have provided complementary insights into body burden and exposure. Future studies should include both blood and urine samples to better evaluate metal exposure and its association with EC stage.

Another limitation of our study is the potential for reverse causation, as urine samples were collected at the time of hospitalization for EC. Physiological processes associated with late-stage disease, including increased systemic inflammation, altered kidney function, and cancer-related metabolic changes, may have influenced metal metabolism and excretion, leading to higher observed urinary metal levels in late-stage cases compared to early-stage cases.

Additionally, the case series nature of the study limits our ability to establish temporality, as the timing of metal exposure in relation to the development and progression of EC remains unclear. Future research with longitudinal designs, pre-diagnostic sample collection, and the use of multiple biomarkers, such as blood and tissue, is needed to better assess causal relationships and minimize the potential impact of reverse causation on findings.

The primary limitation of the database/samples used in this study is the lack of detailed demographic and lifestyle variables, such as occupational history, dietary habits, or environmental exposures, which could provide critical insights into potential pathways of metal exposure. Additionally, more granular data on residential history, proximity to industrial sites, or sources of contamination (e.g., water or soil) would strengthen the analysis. The inclusion of biomarkers related to exposure pathways, such as oxidative stress markers or hormonal profiles, would also be valuable for understanding the mechanistic links between metal exposure and EC progression. Nevertheless, this study provides critical insight into the effect of toxic metals and essential elements on EC staging. Future research should aim to incorporate these variables to provide a more comprehensive analysis of exposure risks and outcomes.

## 5. Conclusions

This study highlights the intricate relationship between essential elements and toxic metals in the development and progression of EC. Bayesian kernel machine regression (BKMR) and other analytical methods revealed that essential elements like zinc and copper play a protective role, with zinc emerging as the most significant predictor among all variables. Conversely, toxic metals such as cadmium and lead demonstrated a stronger association with advanced EC stages, with cadmium’s classification as a Group I carcinogen further supporting its role in EC risk. Mercury showed minimal influence in our analyses.

The broader implications of these findings underscore the dual nature of essential elements—critical for maintaining physiological homeostasis at optimal levels but potentially harmful in excess. In contrast, toxic metals consistently contribute to oxidative stress, cellular dysfunction, and carcinogenesis, compounding the risk of EC. This study emphasizes the need for continued research into the combined and individual effects of metal exposures on EC, with a focus on elucidating underlying mechanisms such as oxidative stress, metallothionein dysregulation, and estrogen receptor interactions. Understanding these interactions can inform targeted public health interventions, reduce environmental exposures, and potentially mitigate EC risk in vulnerable populations.

## Figures and Tables

**Table 1:** Clinical and Pathological Characteristics of the EC Cohort.

Characteristics	Endometrioid (*n* = 229)	Non-Endometrioid *p*-Value (*n* = 37)
Age (yrs), mean (SE)	60.62 (0.7)	57.99 (2.1) 0.05
BMI, kg/m^2^, mean (SE)	35.78 (0.7)	32.62 (1.4) 0.47
Race, *n* (%)		
White	158 (69.0)	27 (73.0) 0.48
Black	22 (9.6)	5 (13.5)
Hispanic	49 (21.4)	5 (13.5)
Stage Category, *n* (%)		
Early (I or II)	190 (83.0)	13 (35.1) <0.001
Late (III or IV)	39 (17.0)	24 (64.9)
Myometrial Invasion		
Superficial	153 (66.8)	16 (43.2) 0.33
Deep	75 (32.8)	11 (29.7)
None	6 (2.6)	10 (27.0)

*p*-value is significant at <0.05 (*p* < 0.05) and was derived using *t*-test (continuous variable) and chi-square (categorical variables). BMI, body mass index; superficial myometrial invasion is less than 50% of myometrial thickness; deep invasion is 50% or greater myometrial thickness.

**Table 2:** Metal Distribution in Early and Late EC Stages.

Metals	Early Stage (µg/L) (SE)	Late Stage (µg/L) (SE)	*p*-Value
Zn	179.00 (30.00)	213.00 (17.00)	0.8826
Cd	6.00 (1.00)	5.00 (0.40)	0.9983
Hg	10.0 (3.0)	-	N/A
Pb	7.00 (4.00)	4.00 (1.00)	0.1338
Cu	8.00 (1.00)	25.00 (17.00)	0.7165

*p*-value is significant at <0.05 (*p* < 0.05). The *p*-values were derived using *t*-test. Mean (SE). N/A, Not available.

**Table 3:** Linear Regression Analysis of the Association between Elements/Toxic Metals and EC Stage.

Metals	Coefficient	95% CI	*p*-Value
Zn	−0.014	−0.036–0.009	0.233
Cu	−0.008	−0.032–0.016	0.509
Hg	1.874	−0.721–4.469	0.156
Cd	0.794	−0.381–1.969	0.185
Pb	0.147	−0.227–0.521	0.509

*p*-value is significant at <0.05 (*p* < 0.05). Adjusted for age of diagnosis, BMI, and race.

**Table 4:** Posterior inclusion probabilities for the influence of toxic metals and essential elements on EC stage.

Variable	PIP
Zinc	0.326
Copper	0.272
Cadmium	0.152
Mercury	0.084
Lead	0.122

**Table 5:** Hierarchical BKMR-posterior inclusion probabilities for the influence of toxic metals and essential elements on EC stage.

Variable	Group	GroupPIP	CondPIP
Zinc	1	0.655	0.517
Copper	1	0.655	0.483
Cadmium	2	0.457	0.278
Mercury	2	0.457	0.039
Lead	2	0.457	0.682

## Data Availability

The raw data supporting the conclusions of this article will be made available by the authors on request.
